# Maximizing outcomes in the treatment of radial head fractures

**DOI:** 10.1186/s10195-019-0523-5

**Published:** 2019-03-23

**Authors:** Stephanie J. Swensen, Vineet Tyagi, Carlos Uquillas, Rachel J. Shakked, Richard S. Yoon, Frank A. Liporace

**Affiliations:** 1 Division of Orthopaedic Trauma, Department of Orthopaedic Surgery, NYU Hospital for Joint Diseases, New York, NY USA; 2 Division of Orthopaedic Trauma, Department of Orthopaedic Surgery, Yale-New Haven Hospital, New Haven, CT USA; 3 Division of Orthopaedic Trauma and Complex Adult Reconstruction, Department of Orthopaedic Surgery, Jersey City Medical Center at RWJ Barnabas Health, 355 Grand Street, Jersey City, NJ 07302 USA

**Keywords:** Radial head fracture, Radial head replacement, Arthroplasty, Internal fixation, Elbow fracture

## Abstract

The radial head plays a critical role in the stability of the elbow joint and its range of motion. Injuries may occur across a spectrum of severity, ranging from low energy non-displaced fractures to high energy comminuted fractures. Multiple classification systems exist to help characterize radial head fractures and their associated injuries, as well as to guide treatment strategies. Depending on the type of fracture, non-operative management may be possible if early range of motion is initiated. Other options include open reduction and internal fixation or excision followed by arthroplasty. A lateral approach is typically used for adequate surgical exposure. Controversy still remains regarding operative management of more severe fractures, but studies have shown good outcomes after radial head replacement for these fractures. We will review the current treatments available for radial head fractures, highlighting gaps in knowledge, as well as providing recommendations for the care of these injuries.

**Level of evidence:** Level V.

## Introduction

Over the past three decades, our evolving understanding of the elbow has provided great insight into the nuances of a complex diarthrodial joint [2, 4, 24, 27, 31, 32]. Through a synergistic relationship between a bony foundation and its surrounding soft tissue, the radial head is an important component that allows for the inherent stable fluidity that lies in the elbow. Previously considered an expendable skeletal component, studies have countered this concept, as injury or fracture to the radial head can lead to significant deficits and functional limitations [4, 21, 24, 40, 44, 49].

Elbow fractures account for approximately 5% of all sustained fractures [29, 45]. Of those cases, radial head fractures account for about one third, making up a significant portion of elbow injuries [29, 45]. This relatively high incidence rate, along with a mean affected patient age of 45 years, has the potential to negatively impact a large portion of the young, active patient population [37, 40, 45].

Radial head injuries occur on a spectrum, from isolated low-energy minimally displaced fractures to the high-energy impacted, comminuted fracture with associated ligamentous and bony injury. Stiffness, the most common complication seen following radial head injury, often contributes to functional deficits, even in injuries at the less severe end of the spectrum. While early aggressive range of motion (ROM) is often used to avoid stiffness, the ability to allow for early ROM largely depends on the stability of the elbow, especially following operative reconstruction. Optimizing outcomes following radial head fracture requires an intimate understanding of the anatomy and biomechanics and the appropriate management of every clinical situation, especially those in which operative intervention is required.

The focus of this article will offer an evidence-based approach to treatment of radial head fractures. Anatomy, biomechanics and associated clinical presentation and fracture classification will be reviewed, followed by a current update and review of the validated outcomes following either reconstruction or replacement. Finally, our own recommendations will be presented based on the reviewed literature with the hopes of providing treatment pearls to maximize patient outcomes following injury.

## Anatomy

The morphology of the radial head is highly variable. Numerous cadaveric studies have demonstrated that the native radial head is not completely circular, nor does it have a consistently elliptical shape [24, 49]. Additionally, the radial head is variably offset from the axis of the neck [24]. The concave region of the radial head articulates with the reciprocally curved capitellum and the peripheral surface of the rim articulates with lesser sigmoid notch. Despite the extensive morphological heterogeneity, van Riet et al. demonstrated that the orientation of the long axis of the radial head is consistently perpendicular to the radial notch of ulna with the forearm in neutral rotation [49].

Articular cartilage covers the concave surface of the radial head in an approximately 280° arc. The remaining non-articulating 80° on the posterolateral region of the radial head has commonly been deemed the “safe zone” for screw fixation following displaced fractures [7–9]. The zone corresponds to a region between longitudinal lines along the radial styloid and Lister’s tubercle. Visual confirmation of the safe zone is possible since the cartilage is slightly darker and thinner than the thicker, white cartilage of the articulating portion [9].

An understanding of the neurovascular supply to the radial head is essential for operative interventions. The radial head has a dual extraosseous blood supply: direct flow from a single branch of the radial recurrent artery, and additional flow from branches from the radial and interosseous recurrent artery, which penetrate the capsular insertion at the neck of the radius. The perforating vessel enters the non-articular portion of the radial head and therefore may have vascular implications for the placement of fixation devices [10]. The posterior interosseous nerve courses around the radial neck and in close proximity to the radial head. The nerve may be protected from iatrogenic injury by pronating the forearm, dissecting the supinator bluntly, and by avoiding the use of retractors over the radial neck [11].

## Biomechanics

The radial head is an essential contributor to valgus, axial, and posterolateral stability of the elbow [2, 8, 12–20]. The important stabilizing role of the radial head is most pronounced in the setting of ligamentous incompetence, which often occurs in association with radial head fractures [31, 32]. Morrey et al. demonstrated in a cadaveric study that the radial head functions as a secondary stabilizer to valgus stress in the medial collateral ligament-deficient elbow [32]. Further studies have also revealed that the kinematics and valgus stability of the elbow are altered by radial head excision in the setting of intact collateral ligaments [16, 27].

Excision of the radial head has also been found to significantly contribute to posterolateral rotatory instability [16]. These findings are due to the loss of capture of the anterolateral rim of the radial head by the capitellum, as well as decreased tensioning of the lateral collateral ligament (LCL) [2, 16, 27]. With an intact LCL, the radial head is less critical to maintain posterolateral stability, suggesting the importance of LCL repair to maintain overall elbow stability [16, 31, 32].

The radiocapitellar alignment is critical for axial loading and longitudinal stability. Radiocapitellar articulations may transmit up to 60% of loads applied across the elbow [4, 13]. In a biomechanical study, Morrey et al. showed that force transmission is greatest between 0° and 30° of flexion and is greater in pronation than in supination [31]. The load transfer is provided by axial engagement of the radial head against the capitellum in addition to the interosseous membrane, distal radioulnar joint ligaments, and triangular fibrocartilage complex. The radial head and soft tissue structures are also essential primary restraints to proximal migration of the radius [4, 10, 16, 27].

## Clinical presentation

The majority of radial head fractures are the result of a fall onto an outstretched hand. Fractures occur when the radial head impacts the capitellum, which may occur from axial, valgus, or posterolateral rotary forces. A thorough physical exam is essential to identify signs of an acute fracture, as well as elbow or forearm instability. The patient may present with pain and limited elbow or forearm range of motion. Inspection often reveals ecchymosis and swelling along the forearm and medial and lateral elbow. Palpation of the radial head, proximal ulna, distal humerus, medial collateral ligament (MCL), LCL, interosseous ligament, and distal radioulnar joint (DRUJ) should be performed. Elbow range of motion, including flexion, extension, supination, and pronation, must also be carefully evaluated. To determine whether any decreased range of motion is due to mechanical block or pain, arthrocentesis can be performed through a direct lateral approach to remove the hemarthrosis and to inject lidocaine intra-articularly. Elbow stability must also be confirmed by testing valgus-varus laxity and performing pivot-shift test to assess for posterolateral rotary instability [25]. A careful vascular exam and neurologic evaluation of the radial, median, and ulnar nerves must be completed. Special attention should be given to assessment of the posterior interosseous nerve.

Anteroposterior, lateral, and oblique (Greenspan) views are obtained for the identification of radial head fractures and aid in further classification of the injury. The special Greenspan view is taken with the forearm in neutral position and the X-ray beam centered on the radiocapitellar joint. The view allows for clear visualization of the radial head without the overlap of the coronoid. Computed tomography (CT) may be utilized for further characterization of the fracture pattern for preoperative planning. Magnetic resonance imaging (MRI) is rarely necessary, but may be useful to assess for soft tissue injuries associated with radial head fractures. Imaging of additional joints is performed as warranted by the clinical presentation.

## Associated injuries

The severity of radial head fractures correlates strongly with the presence of associated injuries. Incidence of associated injuries increases from 20% in non-displaced fractures to 80% in comminuted radial head fractures [37, 45]. Ring et al. [37] summarized the most common injuries associated with radial head fractures into 5 patterns: (1) fracture of the radial head and rupture of the interosseous ligament of the forearm (Essex-Lopresti) [10]; (2) fracture of the radial head and rupture of the MCL or capitellar fracture; (3) fracture of the radial head and posterior dislocation of the elbow; (4) terrible triad injuries (posterior dislocation of the elbow with fractures of the radial head and coronoid process); and (5) posterior olecranon fracture dislocations (posterior Monteggia pattern injuries). In addition to these common injuries, about 10% of patients with a radial head fracture sustain injuries in locations other than the elbow, including fractures of the hand or wrist and scaphoid fractures [45, 48].

## Classification

Several classification systems for radial head fractures have been proposed and serve to guide treatment strategies. The Mason classification [29] is most commonly utilized and has been subsequently modified by Morrey [3], Johnston [22], and Hotchkiss [16]. Additionally, the Mayo group recently provided a modification of the Mason classification to include associated bony or ligamentous injuries [48]. Although widely used, the Mason classification has been demonstrated to have poor to moderate intra- and inter-observer reliability [12]. Mason’s original classification describes non-displaced fractures (type I), marginal fractures with displacement (≥ 2 mm) (type II), and comminuted fractures involving the entire radial head (type III). Morrey further modified the Mason classification to quantify the extent of articular fragment displacement (> 2 mm) and fragment size (≥ 30% of the articular surface). Johnston added a fourth type to the Mason classification, which describes a radial head fracture associated with the dislocation of the elbow [22].

Hotchkiss modified the classification system to provide management guidelines [16]. According to the Hotchkiss modification, type I fractures are minimally displaced and can be treated conservatively. Type II fractures are displaced ≥ 2 mm or block forearm rotation and can be treated with ORIF (open reduction and internal fixation). Type III fractures are comminuted fractures of the radial head or neck and are treated with excision or arthroplasty.

## Treatment

Several parameters must be taken into account in order to select the best treatment option. These include fracture stability, displacement, articular involvement, and the presence of associated forearm or elbow injuries. Treatment decisions may be guided, but not strictly based on, classification systems. Factors not taken into account by the current classification systems include osteopenia, articular impaction, radiocapitellar malalignment, and metaphyseal bone loss. These parameters help to guide treatment decisions on a patient-by-patient basis.

Non-operative treatment for radial head and neck fractures involves a short period of immobilization followed by early range of motion. Indications include isolated, non-displaced, or minimally displaced fractures without mechanical blocks to range of motion. In this subset of patients, non-operative treatment yields good results in greater than 80% of patients [1, 15]. Currently, non-operative treatment is indicated in fractures with less than 3 mm of displacement [47]. However, fracture stability, preservation of forearm rotation and radiocapitellar alignment should also be considered. Simple fractures with displacement between 2–5 mm may be treated non-surgically or with internal fixation. There are no Level I/II studies available to guide treatment in this uncommon scenario [51].

Surgical treatment options include excision, internal fixation, or excision and arthroplasty. Goals of operative treatment include restoration of elbow stability and forearm rotation, which allow for early postoperative range of motion. If displacement is more than 2 mm (Mason type II) internal fixation may be indicated, along with treatment of concurrent soft-tissue injuries. Among Mason type II fractures, Rineer et al. demonstrated that loss of cortical contact of at least one fragment is a strong predictor of a complex elbow injury [36]. Treatments of Mason type III fractures are more controversial. Non-operative treatments yield unsatisfactory results. Fragment excision is rarely indicated; if fragments involve less than 25% of the surface area of the radial head, they may be removed without replacement if elbow stability is not compromised. In Mason type III fractures where reduction and internal fixation is not feasible, radial head replacement is indicated.

When operative treatment is indicated, a lateral approach is generally utilized. The incision is centered over the lateral epicondyle and extends along the mid axial line from the lateral aspect of the humerus to the proximal part of the radius. The Kocher interval between the extensor carpi ulnaris and anconeus is commonly employed [8]. This is an extensile approach in which the interval can be extended both distally and proximally as needed. The forearm must be kept pronated to protect the posterior interosseous nerve (PIN). Tornetta et al. found that pronation of the forearm with blind subperiosteal dissection for plate placement does not place the PIN at significant risk for structural injury [46]. Care must be taken to avoid violating the lateral ulnar collateral ligament (LUCL), which lies below the equator of the capitellum. Violation of the LUCL may destabilize the elbow. The Kaplan direct lateral approach, employing the interval between extensor digitorum communis (EDC) and extensor carpi radialis brevis may also be used [23]. This approach will not disrupt the LUCL, as this approach is more anterior. Since it is a more anterior approach, this may put the PIN at risk. However, the Kaplan approach is not an extensile one. Hotchkiss described splitting the EDC to approach these fractures [16]. This approach is anterior to the LUCL complex and there is a low risk of violating these structures. Schimizzi et al. concluded that when using the EDC splitting approach, the PIN is generally safe when dissecting up to 29 mm from the radiocapitellar joint and up to 42 mm from the lateral epicondyle with the forearm in pronation [42]. The PIN is closer to the radiocapitellar joint when the forearm is in supination and neutral, compared to pronation. If posterior or medial exposure is necessary, a posterior midline incision with elevation of full thickness flaps medially and laterally may be employed. Alternatively, resection of the comminuted radial head may allow for access to a coronoid fracture. A separate medially based approach may be used for coronoid fracture fixation if needed [8].

## Techniques and outcomes

### Open reduction and internal fixation (ORIF)

Open reduction and internal fixation (ORIF) of fractures of the radial head revolves around basic principles of articular fracture care: stable, rigid articular surface fixation and restoration of articular congruencies and normal head-neck alignment. During the approach, it is essential that the LUCL is not damaged. This can be avoided by dissection anteriorly to the head and neck. At the time of surgery, examination of the LUCL for injury and instability is necessary. The anterior surface of the lateral epicondyle is exposed to achieve full visualization of the articular surface. Forearm pronation is used to help aid in visualization as well as for protection of the PIN.

A temporary reduction may be obtained for simple, partial articular fractures with Kirschner wires or fracture reduction clamps. Arthroscopic-assisted reduction techniques for the radial head have been described; however, these techniques remain experimental [39, 50]. Small headless compression screws or screws countersunk beneath the articular surface are often used for fixation. Iacobellis et al. treated 32 patients with radial head fractures with Herbert (12 cases) or Osteomed (20 cases) cannulated screws [17]. In this study, 25 patients had Mason type II and 3 had Mason type III fractures. Fixation with screws was found to be effective in non-comminuted fractures of the radial head. Low-profile periarticular plates are used for unstable extra-articular radial neck fractures or combined radial head-neck fractures to secure the head to the neck. The plates are applied in the non-articular previously described “safe zone” of the radial head. Ikeda et al. reported on ten patients with severely comminuted fractures of the radial head (3 Mason type III and 7 Mason type IV) using low-profile mini-plates [20]. At mean follow-up of 28.5 months, all fractures had united and the outcome was excellent in three patients, good in six, and fair in one. Smith et al. [43] described an alternative to plate fixation in comminuted, displaced radial head fractures using obliquely oriented, countersunk screws from the proximal radial head into the proximal neck with good functional outcomes and improved range of motion compared with plate osteosynthesis; however, the results were not statistically significant.

For comminuted fractures in which adequate reduction is difficult to obtain, the “on-table” reconstruction technique may be used. This technique involves removal of the comminuted fragments for reconstruction on the table, outside of the patient, prior to re-introduction and fixation to the neck. Businger et al. evaluated the value of this “on-table” technique in six patients with severely comminuted radial head fractures (2 Mason type III and 4 Mason type IV) [6]. After mean follow-up of 112 months, one patient had symptoms of degenerative changes, but there were no radiographic signs of devitalization at final examination.

Controversy regarding the advantage of surgical treatment of these fractures still exists. A summary of the most relevant recent literature is available in Table 1. Simple, partial articular fractures with greater than 2 mm displacement (Mason type II) have had excellent long-term results with non-operative treatment. Lindenhovius et al. reported on the long-term outcome of operatively treated Mason type II radial head fractures in sixteen patients [28]. These fractures were not associated with dislocation of the proximal forearm or elbow. Patients were evaluated at an average of 22 years after open reduction and internal fixation with screws (11 patients) or a plate and screws (5 patients) and demonstrated no appreciable advantage over the long-term results of non-operative treatment of these fractures. Excision of the radial head has also been used for Mason type II fracture management. Zarattini et al. retrospectively reviewed 59 patients with Mason type II fractures [52]. Twenty-four patients were treated with radial head excision and 35 were treated with open reduction and internal fixation. At average follow-up of 157 months and 125 months, respectively, patients treated with open reduction and internal fixation had less residual pain, greater range of motion, and better strength than patients treated with radial head excision. Additionally, patients treated with fixation had a lower incidence of severe posttraumatic arthritis. As mentioned previously, certain fracture patterns are more amenable to management with open reduction and internal fixation. Ring et al. retrospectively analyzed 56 patients in whom an intra-articular radial head fracture was treated with open-reduction and internal fixation [38]. Thirty patients had a Mason type II fracture and twenty-six had a Mason type III fracture. Out of 14 patients with 3 or more parts, 3 had failure of fixation, 6 had non-union requiring excision, and only 1 had a satisfactory result. Their study suggests that open reduction and internal fixation is best reserved for minimally comminuted fractures with three or fewer articular fragments.

**Table 1:** Summary of studies

Study	N total	Level of evidence	Outcome measure	Outcome score average	Follow-up (mean)	Mason type	Complications
Ring et al. [38]	56	Prognostic II	SBM	92	48	II-38 III-26	Nonunion
Iacobellis et al. [17]	32	Prognostic II	DASH MEPS	Type II-6.8 Type III-9.5 Type II-98.4 Type III-99.3	47	II-25 III-7	Heterotopic ossification (type II-1, III-3)
Nalbantoglu et al. [33]	25	Prognostic II	SBM	Type III-88 Type III w/dislocation-87	27	III-25	Heterotopic ossification (type III w/dislocation-3)
Ikeda et al. [20]	10	Prognostic II	SBM	90.7	28.5	III-3 IV-7	None
Lindenhovius et al. [27]	13	Therapeutic III	ASES MEPS SBM	91 92 91	17 years	II-5 III-8	Early hardware loosening
Ruan et al. [41]	8	Therapeutic III	SBM	12.5% satisfactory	14	III-8	Heterotopic ossification, nonunion and bone absorption
Ikeda et al. [19]	13	Therapeutic III	SBM ASES	90.7 94.6	36	III-3 IV-10	None
Zarattini et al. [52]	35	Therapeutic III	SBM DASH	95.09 2.81	125	II-35	Intraarticular screw placement-1
Chen et al. [7]	23	Therapeutic I	SBM	72.4, 65.2% satisfactory	2.8 years	III-23	Heterotopic ossification-2 Deep wound infection-1 ROM deficit > 30 deg-2
Businger et al. [6]	6	Therapeutic III	SBM MEPI DASH PCS-12 MCS-12	97 99.2 1.94 52.2 59.5	112	III-2 IV-4	Persistent pain requiring resection- 1
Lindenhovius et al. [28]	16	Therapeutic III	SBM MEPI ASES DASH	91 89 93 12	22 years	II-16	Deep infection-1 Superficial infection-1 Transient PIN palsy-1 Restricted forearm ROM-2 Persistent pain-1
Moro et al. [30]	24	Prognostic II	SF-36 DASH MEPI	47 17 80	39	III- 10 IV- 15	None
Grewal et al. [11]	26	Therapeutic IV	SF-36 Physical Mental DASH MEPI ASES Pain Function Satisfaction PREE	41.8 51.5 24.4 80.5 13.4 27.5 9.0 22.9	24.5		Heterotopic ossification Stage I-4 Stage II-1 Stage IV-1
Ruan et al. [41]	14	Therapeutic III	SBM	92.9% satisfactory	15.9	III-14	Heterotopic ossification-3
Chen et al. [7]	22	Therapeutic I	SBM	92.1, 91% satisfactory	2.8 years	III-22	ROM deficit > 30 deg-2 Stiffness caused by prosthesis-1
Iftimie et al. [18]	27	Therapeutic IV	MEPS DASH VAS	96.4 4.89 0.48	16.9 years	II-5 III-16 IV-6	None
Ricon et al. [35]	28	Therapeutic IV	MEPS	92	32	III-28	Ulnar nerve neuropathy-1 Radiocapitellar incongruence-1 Device removal-3
Burkhart et al. [5]	17	Therapeutic IV	MEPS DASH	90.83 9.8	106		Prosthetic dislocation-2 Capitellar erosion- 3
Popovic et al. [34]	11	Therapeutic III	SBM	82	32	III-11	Heterotopic ossification Class IIA-1
Harrington et al. [14]	20	Therapeutic IV	SBM	88	12.1 years	II-1 IV-13	Persistent pain- 4 Heterotopic ossification Stage I-3 Stage II-2 Stage III-3
Dotzis et al. [9]	14	Therapeutic IV	MEPS DASH		63	III-6 IV 8	Severe complex regional pain syndrome

*ASES* American Shoulder and Elbow Surgeons Shoulder Score, *DASH* Disability of Arm Shoulder Hand, *MCS-12* Mental Component Summary, *MEPS* Mayo Elbow Performance Score, *MEPI* Mayo Elbow Performance Index, *PCS-12* Physical Component Summary, *PREE* Patient Rated Elbow Evaluation, *SMB* Broberg and Morrey functional rating score, *SF-36* Short Form-36, *VAS* Visual Analog Scale

Mason type is based on original Mason classification [11]

Complete articular fractures of the radial head (Mason type III) represent a treatment challenge. Fractures with three or more pieces treated with ORIF may develop unpredictable ulnohumeral and forearm motion, as well as failure of fixation, fragment nonunion, and osteonecrosis [38, 41]. However, Nalbantoglu et al. treated 25 patients with comminuted Mason type III fractures and fracture/dislocations with a plate and screws [33]. They found that selected Mason type III radial head fractures and fracture/dislocations could be stabilized satisfactorily with internal fixation. Lindenhovius et al. compared 28 patients with Mason type III fractures treated with either excision (15 patients) or open reduction internal fixation (13 patients) for an average 17-year follow-up [27]. At 1-year follow-up, there were no differences in range of motion in both flexion and rotation arcs. Though ORIF of Mason type III fractures may occasionally fail, it seems to reduce the risk of subsequent elbow dislocation and protect against long-term arthrosis compared to excision. Ikeda et al. similarly found improved results of ORIF compared to excision in their series of 28 patients, with greater strength and better function [19].

### Radial head replacement

Therapeutic Level I evidence exists which supports radial head replacement for Mason type III fractures compared to ORIF. Chen et al. performed a prospective randomized controlled trial for 45 patients with unstable, comminuted fractures of the radial head [7]. At an average 2-year follow-up, radial head replacement with a metal prosthesis resulted in favorable joint function compared with ORIF. Further studies comparing Mason type III radial head fractures treated with internal fixation and radial head replacement showed similar results [26].

The use of metal radial head prosthesis to help stabilize an elbow is appealing because internal fixation of multi-fragment, displaced fractures of the radial head is susceptible to early or late failure. Radial head excision in these cases can lead to instability, especially if the radial head fracture is a component of a more severe injury such as elbow fracture-dislocations or Essex Lopresti lesions. There are several types of metal prostheses that are currently in use. The three types of metal stems include loose stem, press-fit stem, and cemented stems. Loose-stemmed prostheses act as a stiff spacer. The three types of metal heads include circular monopolar, circular bipolar, and anatomic. Bipolar prostheses are cemented into the radial neck and theoretically can provide improved congruency during elbow motion. Complications of current designs are related to overstuffing the joint. This leads to capitellar wear problems and malalignment instability.

Debate over the treatment of Mason type III fractures still exists. Radial head replacement has been associated with improved patient satisfaction, decreased complications, and better results than ORIF. Ruan et al. compared open reduction and internal fixation with radial head replacement for 22 patients with Mason type III fractures [41]. Of these patients, 14 were treated with bipolar metal head prosthesis and 8 were treated with open reduction and internal fixation. Notably, the results were good to excellent in 92.9% of prosthesis replacement patients and only in 12.5% of ORIF patients (*p* = 0.0004). Chen et al. found that patients receiving radial head replacement achieved significantly better clinical results (91% vs 65.2%) and significantly lower complications rates (13.6% vs 47.9%) [7]. Recovery for radial head replacement primarily occurs in the first 6 months, with high patient satisfaction at 3 months even though patients had slight-to-moderate deficits in range of motion and strength compared to the uninjured contralateral side [11].

Loose-stemmed prostheses have similarly had encouraging results for treating comminuted radial head fractures, with or without associated elbow instability. Moro et al. reviewed 25 patients with displaced, unreconstructable fractures of the radial head and found the treatment to be safe and effective at short-term follow-up [30]. Popovic et al. performed a prospective review of eleven patients with a loose stemmed implant and found good-to-excellent results in eight of the eleven patients with mean follow up of 32 months [34]. In medium-term follow-up (mean 5.3 years), Dotzis et al. found that none of the 14 patients treated with a floating radial head prosthesis had secondary instability of the elbow, implant loosening, cubitus valgus, osteoporosis of the capitellum, or pain in the forearm or wrist [9].

In a long-term study, Burkhart et al. showed that bipolar radial head arthroplasty is associated with good clinical results despite the development of radiographic signs of degenerative arthritis after 8.8 years [5]. Harrington et al. performed a long-term review of 20 patients with mean follow up of 12.1 years [14]. They found results that were excellent in 12 patients, good in 4 patients, fair in 2 patients, and poor in 2 patients. Their results suggest that this construct functions well on a long-term basis. Silicone prostheses are no longer recommended as replacements. They lack the necessary biomechanical support for the elbow joint. Metal prostheses have become commonplace as a result. As an alternative to metal prostheses, Ricon et al. examined twenty-eight patients treated with pyrocarbon prostheses for Mason type III fractures [35]. The results were considered good-to-excellent in 25 patients, with a high degree of satisfaction and functional restoration.

In fractures with associated elbow joint dislocation or forearm interosseous ligament failure, resection is contraindicated due to inherent instability. However, in patients with Mason type III radial head fractures with intact ligaments, resection is still an option. Iftimie et al. showed that at long-term follow-up (mean 17 years), resection arthroplasty offers satisfactory functional results in 96% of patients [18]. In their series of 27 patients treated with excision of the radial head, 26 patients had good-to-excellent results with a mean range of motion of 5°–135°, despite finding osteoarthritic changes in 24 patients.

## Surgical pearls

Several key principles are essential for successful surgical management of radial head fractures. Stability of the elbow is of paramount consideration. The authors suggest a low threshold to replace the radial head in cases of comminuted radial head fractures, given the good functional outcomes and low complication rate with these implants. When performing replacement it is important to appropriately size the implant to balance the need for joint stability with the risk of overstuffing the joint. We recommend biplanar intraoperative fluoroscopic evaluation of the radiocapitellar joint to ensure proper alignment, in conjunction with range of motion and stability testing. Additionally, the necessity of evaluation of the medial side of the elbow after fixation or replacement of the radial head must not be underestimated. Medial elbow pathology, particularly coronoid fractures, should be addressed if there is persistent instability. Anteromedial coronoid fractures frequently require direct visualization and fixation, and should be identified and considered preoperatively. An overhead motion protocol is the preferred rehabilitation after the management of these injuries to maximize range of motion and functional outcomes while minimizing instability.

## Conclusion

Due to the relatively high frequency of radial head fractures, the high incidence of associated injuries, and the variations in treatment, fractures of the radial head continue to present challenges to the orthopaedic surgeon. The surgeon must assess all aspects of the fracture, including stability, comminution, articular depression, and associated injuries of the elbow and forearm during the decision making process. Current evidence supports open reduction and internal fixation of simple Mason type II fractures. For Mason type III fractures, controversy still exists regarding the optimal treatment. Studies currently show good-to-excellent results with radial head replacement in the majority of patients with Mason type III fractures with three or more fracture fragments.

## References

[1] Akesson T, Herbertsson P, Josefsson PO (2006). Primary nonoperative treatment of moderately displaced two-part fractures of the radial head. J Bone Joint Surg Am.

[2] Beingessner DM, Dunning CE, Gordon KD (2005). The effect of radial head fracture size on elbow kinematics and stability. J Orthop Res.

[3] BF M (1985) Radial head fractures. In: BF M (ed). The elbow and its disorders. WB Saunders, Philadelphia, p 355

[4] Bryce CD, Armstrong AD (2008). Anatomy and biomechanics of the elbow. Orthop Clin North Am.

[5] Burkhart KJ, Mattyasovszky SG, Runkel M (2010). Mid- to long-term results after bipolar radial head arthroplasty. J Shoulder Elbow Surg..

[6] Businger A, Ruedi TP, Sommer C (2010). On-table reconstruction of comminuted fractures of the radial head. Injury.

[7] Chen X, Wang SC, Cao LH, Yang GQ, Li M, Su JC (2011). Comparison between radial head replacement and open reduction and internal fixation in clinical treatment of unstable, multi-fragmented radial head fractures. Int Orthop.

[8] Cheung EV, Steinmann SP (2009). Surgical approaches to the elbow. J Am Acad Orthop Surg.

[9] Dotzis A, Cochu G, Mabit C (2006). Comminuted fractures of the radial head treated by the Judet floating radial head prosthesis. J Bone Joint Surg Br.

[10] Essex-Lopresti P (1951). Fractures of the radial head with distal radio-ulnar dislocation; report of two cases. J Bone Joint Surg Br.

[11] Grewal R, Macdermid JC, Faber KJ (2006). Comminuted radial head fractures treated with a modular metallic radial head arthroplasty. Study of outcomes. J Bone Joint Surg Am.

[12] Guitton TG, Ring D, Science of Variation G (2011). Interobserver reliability of radial head fracture classification: two-dimensional compared with three-dimensional CT. J Bone Joint Surg Am.

[13] Halls AA, Travill A (1964). Transmission of pressures across the elbow joint. Anat Rec.

[14] Harrington IJ, Sekyi-Otu A, Barrington TW (2001). The functional outcome with metallic radial head implants in the treatment of unstable elbow fractures: a long-term review. J Trauma.

[15] Herbertsson P, Josefsson PO, Hasserius R (2004). Uncomplicated Mason type-II and III fractures of the radial head and neck in adults. A long-term follow-up study. J Bone Joint Surg Am..

[16] Hotchkiss RN (1997). Displaced fractures of the radial head: internal fixation or excision?. J Am Acad Orthop Surg.

[17] Iacobellis C, Visentin A, Aldegheri R (2012). Open reduction and internal fixation of radial head fractures. Musculoskeletal Surg.

[18] Iftimie PP, Calmet Garcia J, Forcada DI (2011). Resection arthroplasty for radial head fractures: long-term follow-up. J Shoulder Elbow Surg..

[19] Ikeda M, Sugiyama K, Kang C (2005). Comminuted fractures of the radial head. Comparison of resection and internal fixation. J Bone Joint Surg Am.

[20] Ikeda M, Yamashina Y, Kamimoto M (2003). Open reduction and internal fixation of comminuted fractures of the radial head using low-profile mini-plates. J Bone Joint Surg Br.

[21] Itamura JM, Roidis NT, Chong AK (2008). Computed tomography study of radial head morphology. J Shoulder Elbow Surg..

[22] Johnston GW (1962). A follow-up of one hundred cases of fracture of the head of the radius with a review of the literature. Ulster Med J.

[23] Kaplan EB (1968). The etiology and treatment of epicondylitis. Bull Hosp Joint Dis.

[24] King GJ, Zarzour ZD, Patterson SD (2001). An anthropometric study of the radial head: implications in the design of a prosthesis. J Arthroplasty.

[25] Lee ML, Rosenwasser MP (1999). Chronic elbow instability. Orthop Clin North Am.

[26] Li N, Chen S (2014). Open reduction and internal-fixation versus radial head replacement in treatment of Mason type III radial head fractures. Eur J Orthop Surg Traumatol.

[27] Lindenhovius AL, Felsch Q, Doornberg JN (2007). Open reduction and internal fixation compared with excision for unstable displaced fractures of the radial head. J Hand Surg.

[28] Lindenhovius AL, Felsch Q, Ring D (2009). The long-term outcome of open reduction and internal fixation of stable displaced isolated partial articular fractures of the radial head. J Trauma.

[29] Mason ML (1954). Some observations on fractures of the head of the radius with a review of one hundred cases. Br J Surg.

[30] Moro JK, Werier J, Macdermid JC (2001). Arthroplasty with a metal radial head for unreconstructable fractures of the radial head. J Bone Joint Surg Am..

[31] Morrey BF, An KN, Stormont TJ (1988). Force transmission through the radial head. J Bone Joint Surg Am.

[32] Morrey BF, Tanaka S, An KN (1991). Valgus stability of the elbow. A definition of primary and secondary constraints. Clin Orthop Rel Res..

[33] Nalbantoglu U, Kocaoglu B, Gereli A (2007). Open reduction and internal fixation of Mason type III radial head fractures with and without an associated elbow dislocation. J Hand Surg.

[34] Popovic N, Gillet P, Rodriguez A (2000). Fracture of the radial head with associated elbow dislocation: results of treatment using a floating radial head prosthesis. J Orthop Trauma.

[35] Ricon FJ, Sanchez P, Lajara F (2012). Result of a pyrocarbon prosthesis after comminuted and unreconstructable radial head fractures. J Shoulder Elbow Surg..

[36] Rineer CA, Guitton TG, Ring D (2010). Radial head fractures: loss of cortical contact is associated with concomitant fracture or dislocation. J Shoulder Elbow Surg..

[37] Ring D (2008). Displaced, unstable fractures of the radial head: fixation vs. replacement—what is the evidence?. Injury.

[38] Ring D, Quintero J, Jupiter JB (2002). Open reduction and internal fixation of fractures of the radial head. J Bone Joint Surg Am..

[39] Rolla PR, Surace MF, Bini A (2006). Arthroscopic treatment of fractures of the radial head. Arthroscopy.

[40] Rosenblatt Y, Athwal GS, Faber KJ (2008). Current recommendations for the treatment of radial head fractures. Orthop Clin North Am.

[41] Ruan HJ, Fan CY, Liu JJ (2009). A comparative study of internal fixation and prosthesis replacement for radial head fractures of Mason type III. Int Orthop.

[42] Schimizzi A, Maclennan A, Meier KM (2009). Defining a safe zone of dissection during the extensor digitorum communis splitting approach to the proximal radius and forearm: an anatomic study. J Hand Surg Am.

[43] Smith AM, Morrey BF, Steinmann SP (2007). Low profile fixation of radial head and neck fractures: surgical technique and clinical experience. J Orthop Trauma.

[44] Smith GR, Hotchkiss RN (1996). Radial head and neck fractures: anatomic guidelines for proper placement of internal fixation. J Shoulder Elbow Surg..

[45] Tejwani NC, Mehta H (2007). Fractures of the radial head and neck: current concepts in management. J Am Acad Orthop Surg.

[46] Tornetta P, Hochwald N, Bono C (1997). Anatomy of the posterior interosseous nerve in relation to fixation of the radial head. Clin Orthop Rel Res..

[47] Van Glabbeek F, Van Riet R, Verstreken J (2001). Current concepts in the treatment of radial head fractures in the adult. A clinical and biomechanical approach. Acta Orthop Belg.

[48] Van Riet RP, Morrey BF (2008). Documentation of associated injuries occurring with radial head fracture. Clin Orthop Relat Res.

[49] Van Riet RP, Van Glabbeek F, Neale PG (2003). The noncircular shape of the radial head. J Hand Surg.

[50] Wang J, Qi W, Shen X (2018). Results of arthroscopic fixation of Mason type II radial head fractures using Kirschner wires. Medicine (Baltimore).

[51] Yoon A, Athwal GS, Faber KJ (2012). Radial head fractures. J Hand Surg.

[52] Zarattini G, Galli S, Marchese M (2012). The surgical treatment of isolated mason type 2 fractures of the radial head in adults: comparison between radial head resection and open reduction and internal fixation. J Orthop Trauma.

